# The enzyme subunit SubA of Shiga toxin-producing *E. coli* strains demonstrates comparable intracellular transport and cytotoxic activity as the holotoxin SubAB in HeLa and HCT116 cells in vitro

**DOI:** 10.1007/s00204-020-02965-2

**Published:** 2021-01-23

**Authors:** Katharina Sessler, Panagiotis Papatheodorou, Fanny Wondany, Maike Krause, Sabrina Noettger, Denise Bernhard, Jens Michaelis, Herbert Schmidt, Holger Barth

**Affiliations:** 1 Institute of Pharmacology and Toxicology, University of Ulm Medical Center, Albert-Einstein-Allee 11, 89081 Ulm, Germany; 2 Institute of Biophysics, Ulm University, Ulm, Germany; 3 Department of Food Microbiology and Hygiene, Institute of Food Science and Biotechnology, University of Hohenheim, Stuttgart, Germany

**Keywords:** Subtilase cytotoxin, Shiga toxin-producing* Escherichia coli* (STEC), Cellular uptake, Intracellular transport, GRP78

## Abstract

The subtilase cytotoxin (SubAB) is secreted by certain Shiga toxin-producing *Escherichia coli* (STEC) strains and is composed of the enzymatically active subunit SubA and the pentameric binding/transport subunit SubB. We previously demonstrated that SubA (10 µg/ml), in the absence of SubB, binds and intoxicates the human cervix cancer-derived epithelial cell line HeLa. However, the cellular and molecular mechanisms underlying the cytotoxic activity of SubA in the absence of SubB remained unclear. In the present study, the cytotoxic effects mediated by SubA alone were investigated in more detail in HeLa cells and the human colon cancer cell line HCT116. We found that in the absence of SubB, SubA (10 µg/ml) is internalized into the endoplasmic reticulum (ER), where it cleaves the chaperone GRP78, an already known substrate for SubA after its canonical uptake into cells via SubB. The autonomous cellular uptake of SubA and subsequent cleavage of GRP78 in cells is prevented by treatment of cells with 10 µM brefeldin A, which inhibits the transport of protein toxins into the ER. In addition, by analyzing the SubA mutant SubA_ΔC344_, we identified the C-terminal SEEL motif as an ER-targeting signal. Conclusively, our results strongly suggest that SubA alone shares the same intracellular transport route and cytotoxic activity as the SubAB holotoxin.

**Supplementary Information:**

The online version contains supplementary material available at 10.1007/s00204-020-02965-2.

## Introduction

Shiga toxin-producing *Escherichia coli* (STEC) strains are enteric bacterial pathogens and when transmitted to humans, they can cause severe enteric diseases ranging from serious diarrhea to hemorrhagic colitis (HC), as well as diverse extraintestinal diseases such as the life-threatening hemolytic-uremic syndrome (HUS) (Nataro and Kaper, 1998). The pathology of STEC disease is mostly attributed to systemic effects of the Shiga toxin (Stx), however certain strains produce an additional toxin, namely the subtilase cytotoxin SubAB. SubAB was first discovered in the isolate 98NK2 of an O113:H21 STEC strain, which caused a HUS outbreak in South Australia 1998 (Paton et al. 2004; Paton and Paton, 2005). In contrast to other HUS-causing STEC serotypes, this strain lacks the locus of enterocyte effacement (LEE), a well characterized pathogenicity factor causing attaching and effacing lesions in the human intestinal epithelium, which finally results in microvilli degeneration (Moon et al. 1983; Jerse et al. 1990; Jores et al. 2004). Later, SubAB has been detected in strains of various other LEE-negative STEC serotypes (Michelacci et al. 2013; Sánchez et al. 2013). It was shown that SubAB is lethal for mice and induces apoptosis in human renal tubular epithelial cells (Paton et al. 2004; Márquez et al. 2014), suggesting that SubAB might be a relevant pathogenicity factor in STEC infections.

SubAB belongs to the family of AB_5_ toxins, consisting of an enzymatically active A subunit (SubA) and separate B subunits (SubB). SubB forms pentamers, binds to the cell surface and is responsible for the uptake of SubA into the host cell (Paton et al. 2004). SubB binds preferred to glycans terminating in the sialic acid *N*-glycolylneuraminic acid (Neu5Gc) (Byres et al. 2008). Additionally, it was shown that glycans terminating in *N-*acetylneuraminic acid (Neu5Ac), *N*-glycans as well as *O*-glycans, and several glycoproteins, including integrin and L1 cell adhesion molecule (L1CAM), serve as SubB-receptors (Yahiro et al. 2006, 2011; Yamaji et al. 2019). Inside the host cell, SubAB is transported via a retrograde pathway into the endoplasmic reticulum (ER). In the ER the enzymatically active A subunit cleaves the glucose-regulated protein GRP78, a chaperone that is also known as immunoglobulin heavy-chain binding protein (BiP) (Paton et al. 2006). SubA-catalyzed GRP78 cleavage leads to an accumulation of unfolded proteins in the ER, triggering the unfolded protein response (UPR) and ultimately causing cell death. SubA acts as a subtilase-like serine protease with a “catalytic triad” of aspartic acid 52, histidine 89, and serine 272, which is responsible for the proteolytic activity (Paton et al. 2004). The exchange of serine 272 to an alanine residue results in an enzymatically inactive SubA, meaning that SubA_S272A_B shows no toxicity in vitro and in vivo (Talbot et al. 2005; Paton et al. 2006).

In a previous study, Western blot analysis revealed that SubA, in the absence of SubB, binds to human cervix cancer-derived epithelial cells (HeLa) and that SubA, in higher concentrations than required for cytotoxic effects of SubAB, exhibits cytotoxicity (Funk et al. 2015). So far, the underlying cellular and molecular mechanisms by which SubA mediates its cytotoxic effects are completely unknown and were aimed to be characterized in the current study. We demonstrated that also human colon epithelial cells (HCT116), as a medically more relevant cell line for an intestinal pathogen, are susceptible to SubA. Confocal fluorescence microscopy revealed that SubA alone is internalized into HeLa cells and co-localizes with ER structures, which strongly suggests that SubA, in the absence of the transporter subunit SubB, reaches the same target compartment as SubAB. SubA, when applied to cells in the absence of SubB, cleaves GRP78, which is inhibited by brefeldin A (BFA). Hence, it can be concluded that SubA alone is taken up into cells via a retrograde pathway like it is known for the holotoxin SubAB. SubA does not contain a classical KDEL motif at its C-terminus such as cholera toxin which ensures retrograde toxin transport (Lencer et al. 1995), however, the C-terminal SEEL motif is predicted as a potential ER-targeting signal by the scanProsite tool (Castro et al. 2006). Therefore, a SubA mutant lacking the C-terminal SEEL motif was generated (SubA_ΔC344_) and its cytotoxic potential on cultured cells was analyzed. SubA_ΔC344_ alone neither showed morphological changes nor GRP78 cleavage on HeLa and HCT116 cells, whereas in combination with SubB it behaved like wildtype SubAB, assuming that this C-terminal SEEL motif is only essential for ER transport of SubA alone. It should be noted that in this study, only the His-tagged subunits of the variant SubAB2-2 were used. Overall, the data contribute to a better understanding about the cell entry routes and mode of action of the subtilase cytotoxin.

## Results

**Fig. 1:**
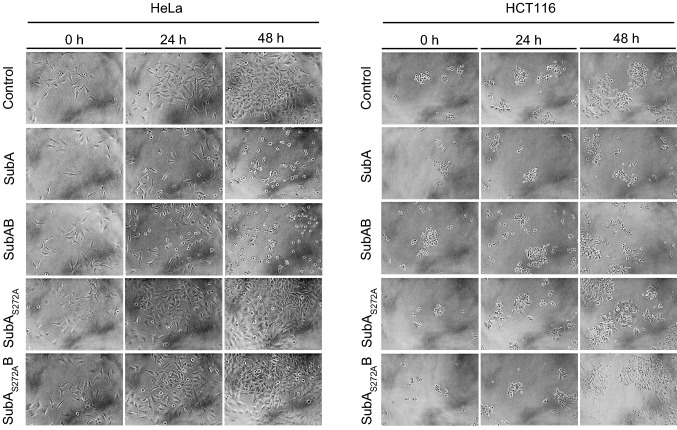
Cytotoxic effects of SubA2-2-His, SubAB2-2-His, SubA2-2-His_S272A_ and SubA2-2His_S272A_B on HeLa and HCT116 cells. Cells were incubated with either 10 µg/ml of the single toxin component or a total protein concentration of 10 µg/ml for both toxin components in a molar ratio of 1:5 for 48 h at 37 °C. For control, cells were left untreated. To demonstrate changes in the cell morphology as well as in the cell number, images were taken after 0, 24, and 48 h. Experiments were performed in triplicates and representative images selected

The cytotoxic effects of SubA in the absence of SubB have already been described for HeLa and Vero cells (Funk et al. 2015). Here, we have additionally examined whether human colon cancer-derived epithelial cells (cell line HCT116) are target cells for the cytotoxic effects caused by SubA alone, since these cells represent, for an intestinal pathogen, a more physiological cell line to investigate the autonomous effects of this toxin component. The cells were treated with 10 µg/ml SubA, 10 µg/ml enzymatically inactive SubA_S272A_, 10 µg/ml SubAB, 10 µg/ml SubA_S272A_B, or were left untreated for control and the cytotoxic effects were analyzed in terms of morphological alterations. As shown in Fig. 1, treatment of cells with SubA alone for 48 h resulted in an obvious change in the morphology of HCT116 cells as well as HeLa cells, which were included in this experiment as a positive control for a SubA susceptible cell line. This change in cell morphology represents an established specific and selective endpoint to monitor the cytotoxic effects of SubAB or SubA in cell-based experiments (Funk et al. 2015). Moreover, treatment of cells with SubA led to a decreased number of cells after 48 h compared to the negative control (without toxin).

**Fig. 2:**
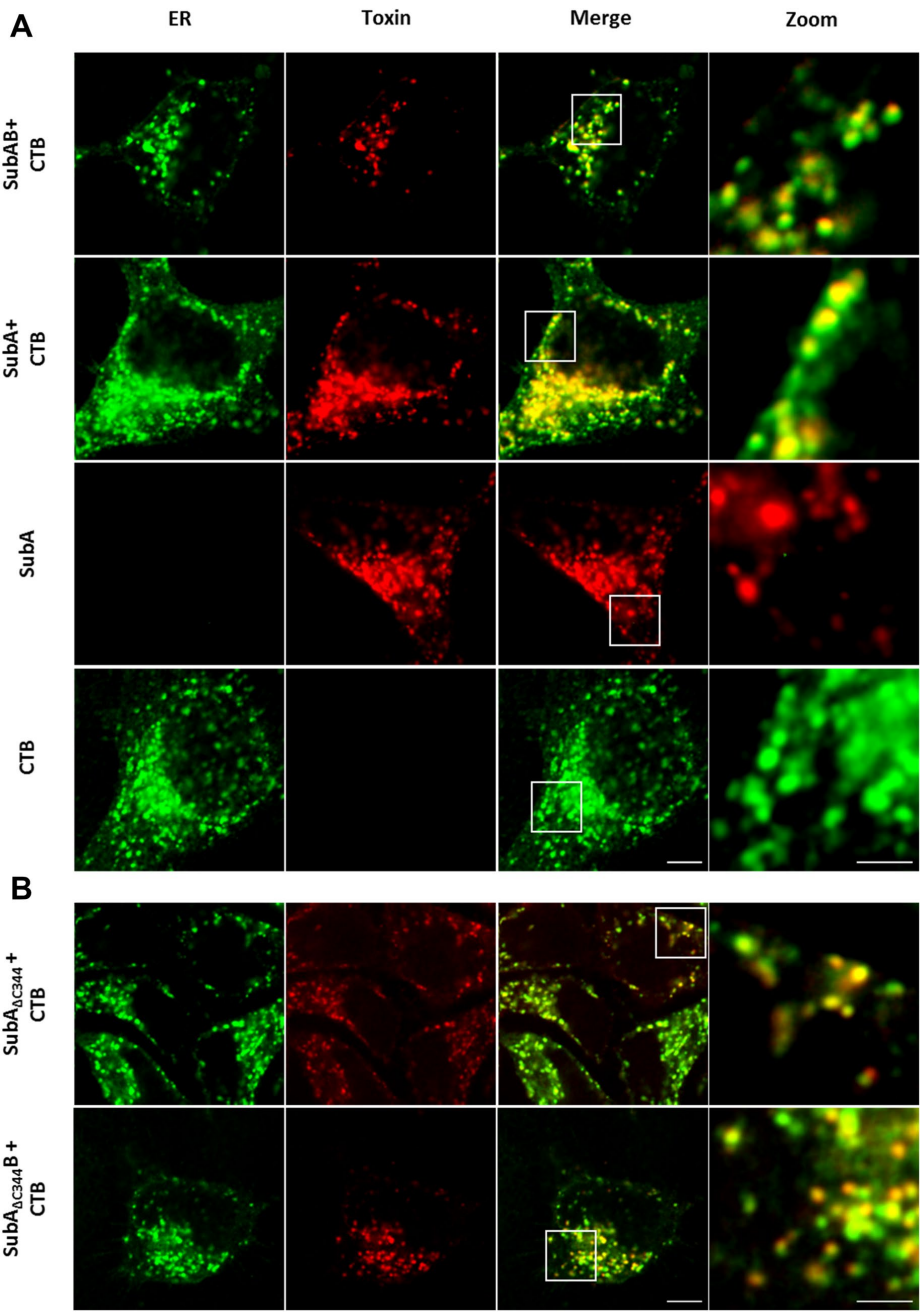
**a** Confocal microscopic images of SubA2-2-His (red) co-localization with the ER (green) in HeLa cells. **b** Confocal microscopic images of SubA_∆344_2–2-His (red) co-localization with the ER (green) in HeLa cells. Cells were incubated for 6 h at 37 °C with 30 µg/ml ATTO647N-labeled SubA2-2-His (indicated as SubA), ATTO647N-labeled SubA_∆344_2–2-His (indicated as SubA_∆344_) or with 20 µg/ml SubA_ATTO-647N_B2-2His (indicated as SubAB) or with SubA_∆344,ATTO-647N_B2-2His (indicated as SubA_∆344_B). The same cells were incubated with 15 µg/ml ALEXAFluor594-conjugated Cholera toxin B subunit (CTB) as established ER marker. In parallel, cells were only incubated with 30 µg/ml ATTO647N-labeled SubA2-2-His or ALEXAFluor594-conjugated CTB. Cells were then washed with PBS, fixed, blocked, and CTB (indicating localization of the ER) and the distribution of the labeled SubA2-2-His was captured via confocal microscopy. Scalebar: 5 µm (Zooms: 2 µm)

**Fig. 3:**
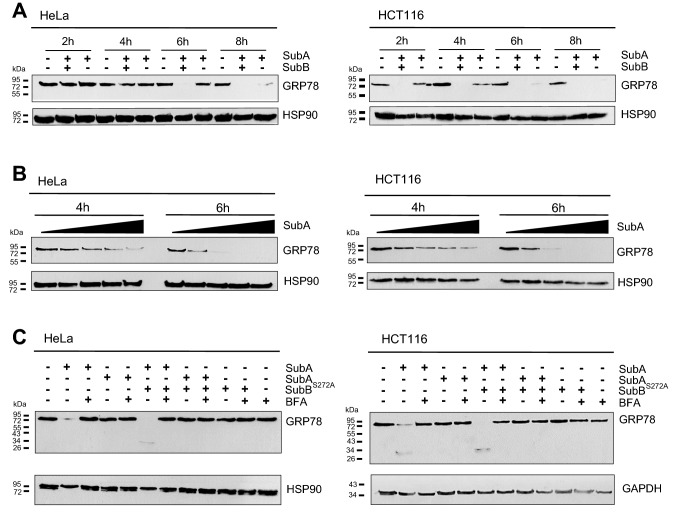
Western blot analysis of GRP78 cleavage due to SubA2-2-His treatment on HeLa and HCT116 cells. Cells, seeded in a 12-well plate, were incubated with or without toxin in FCS-free medium at 37 °C, as indicated. Cells were then solubilized in Lämmli sample buffer and subjected to SDS-PAGE. The substrate status was analyzed by Western blotting using monoclonal mouse anti GRP78 (see Methods). **a** Time course of GRP78 cleavage triggered by 5 µg/ml of either SubAB2-2-His (in a molar ratio of 1:5) or SubA2-2-His alone. **b** Time and concentration course of GRP78 cleavage. The following concentrations of SubA2-2-His, plotted from low to high, were used: 3.4 µg/ml, 6.8 µg/ml, 10.2 µg/ml, 13.6 µg/ml, and incubated for 4 h and 6 h. **c** Effect of brefeldin A (BFA) on GRP78 cleavage. Cells were pre-treated with or without 10 µM BFA and then treated with or without 10 µg/ml SubAB2-2-His (in a molar ratio of 1:5), SubA_S272A_B2-2-His, SubA2-2-His, SubA_S272A_2-2-His_,_ or SubB2-2-His for 4 h at 37 °C

Based on the results obtained so far, the question arose whether SubA mediates its cytotoxic effects via extracellular or intracellular mechanisms. To address this question, the uptake of SubA in the absence of SubB was investigated with a home-built dual-color microscope in confocal mode (Osseforth et al. 2014). HeLa cells were incubated with ATTO647N-labeled SubA (30 µg/ml) and/or the AlexaFluor594-conjugated B-subunit of the cholera toxin (CTB), which was used as an established and specific marker for the ER. For control, HeLa cells were incubated with the combination of SubAB in a final protein concentration of 20 µg/ml in total (molar ratio of 1 (ATTO647N-labeled SubA to: 5 (SubB)). The merged images in Fig. 2 clearly show co-localization of the ER with SubA when SubA was applied to the cells in combination with SubB but interestingly, also, when SubA was applied alone. This implicates that SubA, even without its translocation subunit SubB, reaches the same target compartment as SubAB in cells. To support the hypothesis that SubA is taken up into the ER, when applied in the absence of SubB, we examined GRP78 cleavage in cells via Western blotting. Figure 3a clearly demonstrates that SubA applied without SubB cleaves GRP78 in the cells, however, this cleavage was slower than GRP78 cleavage by SubAB. Comparing HeLa and HCT116 cells, it is noticeable that in HCT116 cells SubAB-catalyzed GRP78 cleavage occurs already after 2 h whereas in HeLa cells it occurs after 6 h. HCT116 cells, compared to HeLa cells, show an earlier response with regards to GRP78 cleavage to SubAB which also applies for SubA induced GRP78 cleavage. Moreover, Fig. 3b shows that SubA-catalyzed GRP78 cleavage occurs in a time- and concentration-dependent manner in HeLa as well as in HCT116 cells. Finally, the results strongly suggested that SubA alone follows the same route into cells as SubAB. To investigate that in more detail, cells were pre-incubated with BFA, which induces the breakdown of the Golgi apparatus and interrupts the retrograde transport pathway to the ER, prior to toxin treatment. Figure 3c demonstrates that pretreatment of cells with BFA inhibited not only the SubAB- but also the SubA-mediated GRP78 cleavage in HeLa as well as in HCT116 cells. This result implicates that SubA alone is also transported via a retrograde pathway into the ER, just like it occurs in combination with SubB.

**Fig. 4:**
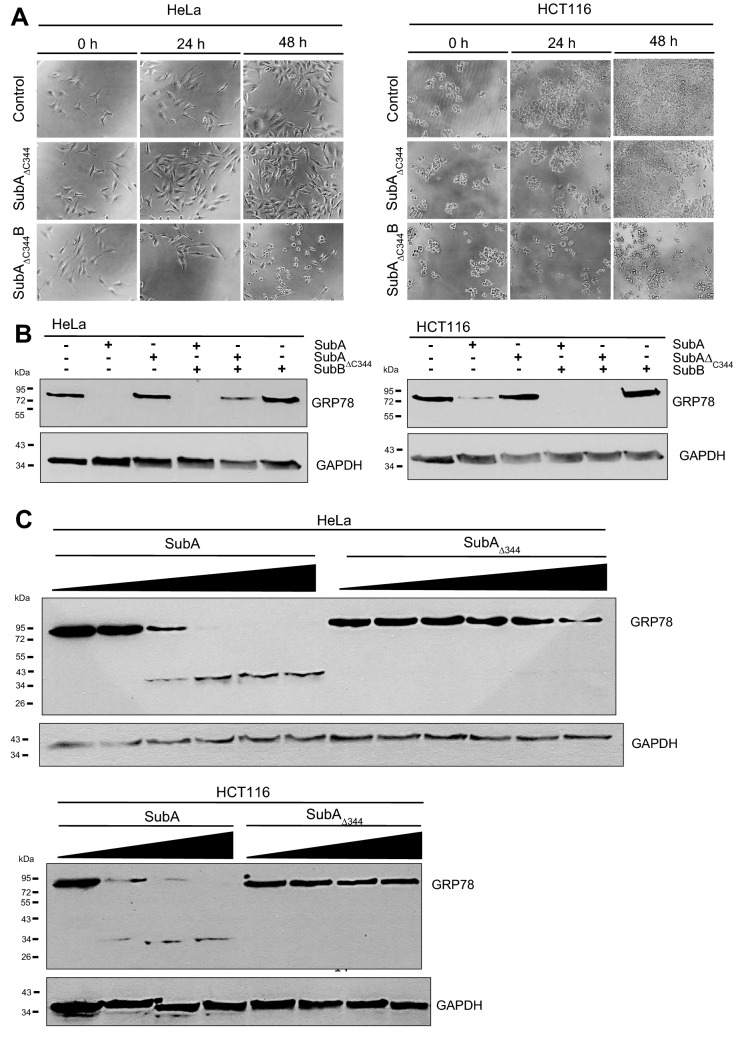
**a** Cytotoxic effects of 10 µg/ml SubA_ΔC344_2-2-His or 10 µg/ml SubA_ΔC344_B2-2-His (in a molar ratio of 1:5) on HeLa and HCT116 cells. Cells were incubated for 48 h at 37 °C. For control, cells were left untreated. To demonstrate changes in the cell morphology as well as in the cell number, images were taken after 0, 24, and 48 h. **b**, **c** Western blot analysis of GRP78 cleavage after treatment with SubA_ΔC344_2-2-His. For **b** cells were seeded in 12-well plates and incubated with 10 µg/ml SubA_ΔC344_2-2-His or a total protein concentration of 10 µg/ml (molar ratio of 1:5) for SubA_ΔC344_B2-2-His in FCS-free medium for 4 h at 37 °C. For **c** cells were seeded in 12-well plates and incubated with increasing concentrations of SubA2-2-His and SubA_ΔC344_2-2-His in FCS-free medium for 6 h at 37° C. The following concentrations of SubA2-2-His and SubA_ΔC344_2-2-His, plotted from low to high, were used: HeLa cells: 2.5 µg/ml, 10 µg/ml, 20 µg/ml, 30 µg/ml, 40 µg/ml; HCT cells: 10 µg/ml, 20 µg/ml, 40 µg/ml. Cells were then solubilized in Lämmli sample buffer and subjected to SDS-PAGE. The substrate status, i.e. GRP78 cleavage, was analyzed by immunoblotting using monoclonal mouse anti GRP78 (see Methods)

Next, we wondered how SubA reaches the ER in the absence of SubB. Obviously, a yet unknown ER targeting signal is present in SubA. Most likely, a C-terminally SEEL-motif which is predicted by the software scanProsite tool might represent an ER-targeting signal. We therefore decided to generate a SubA mutant lacking the C-terminal SEEL motif (SubA_∆C344_). First, we examined the C-terminally truncated SubA_ΔC344_ mutant with regards to its cytotoxicity. The cells were treated with 10 µg/ml SubA_ΔC344_, 10 µg/ml SubA_ΔC344_B, or were left untreated for control. Morphological changes of the cells were photographically documented after 0 h, 24 h, and 48 h. In contrast to the wildtype (shown in Fig. 1), the mutant SubA_ΔC344_ alone did neither cause morphological changes nor a decrease of the amount of HCT116 or HeLa cells (Fig. 4a). Thus, SubA_ΔC344_ alone behaved like the enzymatically inactive SubA_S272A_. Interestingly, SubA_ΔC344_ showed again cytotoxic effects in combination with SubB, indicating that SubA_ΔC344_ remains enzymatically active also with shortened C-terminus. Moreover, an enzyme activity test with cell lysates of HeLa and HCT116 cells proved that only the mutant SubA_S272A_ is enzymatically inactive (see Supplementary Information). We also looked at the binding capacity of SubA_ΔC344_ compared to the wildtype SubA via flow cytometry and determined that both proteins bind to a similar extent to HeLa cells (see Supplementary Information). Next, we examined the uptake of SubA_ΔC344_ into cells. Fluorescence microscopy revealed that SubA_ΔC344_ is taken up by HeLa cells (Fig. 2b), however, this uptake is much less efficient compared to the wildtype SubA (Fig. 4c). When cells were incubated with the combination of SubB and SubA_ΔC344_, GRP78 was cleaved in the cells comparable to the cleavage after treatment of cells with SubB plus wildtype SubA. This indicates that SubA_ΔC344_ is enzymatically active when delivered into the ER. Hence, we identified the C-terminal SEEL motif as an ER targeting signal and therefore as a crucial segment for SubA-mediated cytotoxicity.

## Discussion

In the present study, a series of cell-based experiments was performed to investigate the recently discovered cytotoxicity caused by SubA in the absence of SubB in more detail. In summary, the data revealed that although the SubB binding/transport subunit was lacking, SubA was internalized into the ER of cells where it cleaved its cellular substrate, the chaperone GRP78. Interestingly, SubA entered the same cellular compartment in the absence and presence of SubB, but uptake was increased in the presence of SubB.

The confocal microscopic analysis clearly revealed that SubA is taken up in cells and remarkably that it reaches the ER even without SubB. The fact that the images show a stronger signal for the A-subunit alone compared to the holotoxin is not due to the fact that SubA is taken up more efficiently without SubB but is rather attributed to the different concentrations used. For the holotoxin, an overall lower concentration was used and it should also be noted that fluorescently labeled SubA and non-labeled SubB were mixed in a molar ratio of 1:5. The Western blot analyses confirmed that SubA alone is taken up into the ER and beyond that, it arrives there enzymatically active. The results show that GRP78-cleavage in living cells by SubA is time and concentration dependent. However, GRP78-cleavage occurs slower compared to SubAB, suggesting that the uptake and/or the transport into the ER of SubA alone may be less efficient. Moreover, the SubA-catalyzed cleavage of GRP78 in cells was inhibited by BFA, strongly suggesting that SubA alone is also transported via a retrograde pathway similar to SubAB. The prevention of GRP78 cleavage in cells by SubAB after treatment of cells with BFA, which inhibits SubAB transport into the ER, implicates that the detected GRP78 in the Western Blot experiments comes from the intracellular GRP78 in the ER. BFA itself had no effect on the cell viability of HeLa and HCT 116 cells (Figure S2). Moreover, SubA_ΔC344_, which lacks the SEEL motif, which was predicted as a potential ER-targeting signal by the scanProsite tool (Castro et al. 2006), did not exhibit cytotoxic effects when applied alone to HeLa and HCT116 cells. However, in combination with SubB, SubA_ΔC344_ induced cytotoxicity in HeLa and HCT116 cells, clearly demonstrating that SubA_ΔC344_ is enzymatically active. Although, SubA_ΔC344_ did not induce morphological changes of HeLa and HCT116 cells, fluorescence microscopic analysis revealed that SubA_ΔC344_ is taken up by HeLa cells and, moreover, co-localizes with the ER. However, the Western blot analysis shown in Fig. 4c clearly demonstrates that SubA_ΔC344_ uptake and transport to the ER were much less efficient compared to wildtype SubA. Based on this observation, we concluded that the C-terminal SEEL motif is crucial for guiding SubA into the ER. However, the findings also showed that SubB contains its own ER targeting signal, as SubB delivers SubA_ΔC344_ into the ER.

This also opens the question, whether the A-subunits of other AB_5_ toxins also may cause toxic effects independently to eukaryotic cells and whether other cells may be targeted by a single toxin component than by the holotoxin. Future research is needed to investigate these questions in more detail, because the observation that SubA alone, without its receptor binding subunits may cause cytotoxic effects, means a basic change in the paradigm, that for cytotoxicity a complete AB toxin complex is needed.

## Experimental procedures

### Expression, purification, and characterization of recombinant proteins

SubAB subunits and SubA variants were expressed and purified as described in Krause et al. (2019). In this study we used the variants SubA2-2-His, SubA2-2-His_S272A_, SubA2-2-His_ΔC344_, and SubB2-2-His. The inactive SubA variant was constructed using side directed mutagenesis on the wild type plasmid. The truncated variant was obtained by selected amplification of amino acid 1–344 of the wild type. All subunits were cloned into expression vectors with a C-terminal His tag under T7 promoter control. The expression was performed in 2YT medium after induction with 250 µM IPTG overnight. Subsequently, the cells were harvested, disrupted, and the cleared lysate applied to Ni–NTA beads. After elution of the His-tagged subunits, they were polished on a Superdex75pg 16/600 running in PBS supplemented with 10% glycerol. All subunits were stored in aliquots at − 70 °C. Secondary structure composition and oligomerization were analyzed with CD spectrometry and size exclusion chromatography as described in Krause et al. 2019. All variants show comparable secondary structure composition to the wild type. The elution volumes of the SubA2-2-His variants represent the expected sizes and oligomerization.

### Cell culture and cytotoxicity assays

HeLa cells were routinely grown in MEM medium supplemented with 10% FCS, 1.5 g/l sodium bicarbonate, 2 mM l-glutamine, 1 mM sodium-pyruvate, 0.1 mM non-essential amino acids and 1% penicillin–streptomycin at 37 °C and 5% CO_2_. HCT116 were cultured in DMEM medium containing the same supplements as described for HeLa cells. For cytotoxicity assays, cells were seeded in a 96 well plate with 2 × 10^3^ cells/well and treated with the indicated amounts of toxin. Cells were incubated with the single toxin subunits and in combination of A and B in a molar ration of 1:5. Pictures were taken after 0 h, 24 h and 48 h of intoxication using a Zeiss Axiovert 40CFL microscope with a Jenoptik ProGres C10 CCD camera.

### Analysis of substrate modification by Western blotting

Cells were seeded in a 12 well with 7 × 10^4^ cells/well and incubated with the appropriated amount of either the single toxin subunit or the combination of both compounds (in a molar ratio of 1:5) for 2 to 24 h, as indicated. For inhibitor experiments, cells were pre-incubated with 10 µM BFA for 30 min prior to intoxication. Cells were then washed with PBS and lysed in 2.5 × Lämmli sample buffer containing DTT. Cell lysates were incubated for 10 min at 95 °C, separated by 12.5% SDS-PAGE, and blotted onto a nitrocellulose membrane. Membranes were blocked by 5% nonfat dry milk in PBS-T (PBS containing 0.1% Tween-20) for 1 h at room temperature. GRP78 was detected using anti-GRP78/BiP monoclonal antibody (BD Biosciences, San Rose, CA USA) diluted 1:1000 in blocking solution, followed by goat anti-mouse IgG (H + L) HRP conjugate (SantaCruz Biotechnology, Dallas, TX USA) diluted 1:10,000 in blocking solution. Primary antibody was incubated at 4 °C overnight and secondary antibody was incubated for 1 h at room temperature. To confirm equal amounts of protein, membranes were stripped and antibodies against HSP90 (1:1000, SantaCruz Biotechnology, Dallas, TX USA) or GAPDH (1:1,000, SantaCruz Biotechnology, Dallas, TX USA) were used. Visualization of the signals occurred by enhanced chemiluminescence (ECL system, EMD Millipore Corporation, Darmstadt, Germany).

### Labeling and confocal fluorescence microscopy

Purified SubA was labeled with ATTO-647N-hydroxysuccinimidyl(NHS)-Ester according to manufacturer’s protocol (ATTO-TEC, Siegen, Germany). AlexaFluor594-conjugated CholeraB was obtained from the manufacturer Thermo Scientific (Thermo Scientific, Waltham, MA USA). HeLa cells were seeded in ibidi 8-well µ-slides (Martinsried, Germany) and incubated with indicated toxin concentrations in FCS-free MEM medium at 37 °C for 6 h. Afterwards, cells were washed with ice-cooled PBS, fixed with paraformaldehyde for 20 min at room temperature, blocked with BSA for 2 h at room temperature, and kept in 2,2′-thiodieethanol (97% solution in PBS, pH 7.5) for imaging. Images were taken with a home-built dual-color 3D-STED microscope in confocal mode (Osseforth et al. 2014). Typically, an average power of 1 mW for each excitation beam, 568 nm and 633 nm, respectively, at a repetition rate of 1 MHz was used. Confocal images were captured at a pixel size of 20 nm and a dwell time of 200 µs. Approximately, 100–200 counts were the typical peak photon number. Images were analyzed in ImageJ 1.52n. A Gaussian blur σ = 1 and an intensity threshold of > 10–20 counts was applied in each channel for better visualization.

### Reproducibility and statistics

All experiments were performed independently and at least three times. Results are shown from representative experiments.

## Supplementary Information

Below is the link to the electronic supplementary material.

Supplementary file1 (DOCX 6316 KB)
