# Supplementary material for: The enzyme subunit SubA of Shiga toxin-producing E. coli strains demonstrates comparable intracellular transport and cytotoxic activity as the holotoxin SubAB in HeLa and HCT116 cells in vitro
Source: Arch Toxicol. 2021 Jan 23;95(3):975–83. doi: 10.1007/s00204-020-02965-2 (PMC7904543; doi:10.1007/s00204-020-02965-2)
Supplement: Supplementary file 1 — Supplementary file1 (DOCX 6316 KB) [file 204_2020_2965_MOESM1_ESM.docx]

Supplementary Material

**The enzyme subunit SubA of Shiga Toxin-Producing *E. coli* strains demonstrates comparable intracellular transport and cytotoxic activity as the holotoxin SubAB in HeLa and HCT116 cells in vitro**

Katharina Sessler^1^, Panagiotis Papatheodorou ^1^, Fanny Wondany^2^, Maike Krause^3^, Sabrina Noettger^1^, Denise Bernhard^3^, Jens Michaelis^2^, Herbert Schmidt^3^, Holger Barth^1#^

^1^Institute of Pharmacology and Toxicology, University of Ulm Medical Center, Ulm, Germany

^2^Institute of Biophysics, Ulm University, Ulm, Germany

^3^Department of Food Microbiology and Hygiene, Institute of Food Science and Biotechnology, University of Hohenheim, Stuttgart, Germany

^#^Correspondence: [holger.barth@uni-ulm.de](mailto:holger.barth@uni-ulm.de)

**Supplementary results:**

**SubA alone induces cytotoxicity and cleaves GRP78 in Vero and CaCo-2 cells**

Since HeLa and HCT116 cells were sensitive to SubA alone, two additional cell lines were tested to exclude a cell line specific effect. Vero and CaCo-2 cells show clear changes in cell morphology after 48 h of SubA treatment, indicating that SubA also triggers cytotoxicity in these cell lines. Moreover, Figure S 1B shows a SubA-induced GRP78 cleavage for both cell lines.

**Brefeldin (BFA) does not reduce viability of HeLa and HCT116 cells**

For inhibitor experiments, cells were pre-incubated with 10 µM BFA for 30 min prior to intoxication and substrate status was analyzed via Western blotting. To investigate whether BFA itself exhibits cytotoxic effects, HeLa and HCT116 cells were incubated with 5 µg/ml, 10 µg/ml, or 20 µg/ml BFA for 6.5 h and cell viability was measured via MTS assay. For control, cells were left untreated. Figure S 2 shows that BFA in the concentrations and incubation times used in this study, does not reduce cell viability.

**An enzyme activity test showed that SubA_∆C344_ is enzymatically active**

Since SubA_∆C344_, when applied alone to HeLa or HCT116 cells, showed no morphological changes, we checked its enzyme activity. From the pictures together with SubB (Figure 4A), it appears already that SubA_∆C344_ must be enzymatically active, nevertheless we additionally performed an enzyme activity test to verify that. Cell lysates of HeLa and HCT116 cells were incubated for 30 min at 37 °C with either 10 µg/ml SubA, 10 µg/ml SubA_∆C344_, 10 µg/ml SubA_S272A_ or left untreated for control. Samples were separated by SDS-PAGE, blotted, and GRP78 substrate status was analyzed. The immunoblotting result clearly shows that SubA and SubA_∆C344_ cleave GRP78, whereas for the enzymatically inactive mutant SubA_S272A_ the GRP78 band is still visible (Figure S 3). This result proves the assumption that SubA_∆C344_ is enzymatically active.

**SubA and SubA_∆C344_ bind to a similar extent to HeLa cells**

To analyze cell surface-binding, cells were incubated with DyLight488-labeled SubA (30 µg/ml) or DyLight488-labeled SubA_∆C344_ (30 µg/ml). Binding was performed at 4 °C for 30 min. Subsequently, the cells were washed once to remove unbound or non-specifically bound protein and the amount of cell-bound fluorescence was measured. The results shown in Figure S 4 revealed that on the one hand SubA alone binds to HeLa cells and on the other hand that SubA_∆C344_ binds to a similar extent like the wildtype.

# Supplementary experimental procedure

**Cytotoxicity assay**

Vero and CaCo-2 cells were seeded in 96-well plates with 2 x 10^3^ cells/well and treated with the indicated amounts of toxin. Cells were incubated with the single toxin subunits A or B, as well as in combination of A and B in a molar ration of 1:5. Pictures were taken after 0 h, 24 h, and 48 h of incubation using a Zeiss Axiovert 40CFL microscope with a Jenoptik ProGres C10 CCD camera.

**Cell viability test**

HeLa and HCT116 cells were seeded in 96-well plates with 7 x 10^4^ cells/well and incubated with 5 µg/ml, 10 µg/ml, or 20 µg/ml BFA for 6.5 h or left untreated for control. Afterwards, MTS substrate was added for 2 h and absorbance was measured at 490 nm via a 96-well micro plate reader.

**Enzyme activity test**

Whole HeLa and HCT116 cell lysates were incubated for 30 min at 37 °C with either 10 µg/ml SubA, 10 µg/ml SubA_∆C344_, 10 µg/ml SubA_S272A_ or left untreated for control. Afterwards 5x Lämmli sample buffer + DTT was added and samples were incubated at 95 °C for 10 min. Samples were then separated by a 12,5 % SDS-PAGE and blotted onto a nitrocellulose membrane. From this point on, the same procedure was performed as described under “Analysis of substrate modification by Western blotting” in the original article.

**Flow cytometry**

Confluently grown cells were detached from culture dishes by applying 25 mM EDTA/PBS. Cell suspension were centrifuged at 500 rpm for 5 min and washed twice using PBS. Cells were kept on ice, counted, and 200,000 cells/sample were incubated for 30 min at 4 °C in PBS with the indicated concentrations of labeled toxin. To get rid of unbound toxin, cells were centrifuged at 500 x g for 3 min and washed once with ice-cooled PBS. Cell surface-bound fluorescence was measured using the BD FACSCelesta platform via an argon-ion laser (488 nm) and the 530 nm band pass filter (FITC). Toxins were labelled with a Dylight488 NHS Ester according to the manufacture´s manual (Thermo Scientific, Waltham, MA USA).

Figure S 1: (A) Cytotoxic effects of SubA2-2-His and SubAB2-2-His on Vero and CaCo-2 cells. Cells were incubated with either 10 µg/ml of the single toxin component (SubA) or a total protein concentration of 10 µg/ml for both toxin components (SubAB) in a molar ratio of 1:5 for 48 h at 37 °C. For control, cells were left untreated. Images were taken after 0 h, 24 h, and 48 h. Experiments were performed in triplicates and representative images were selected. (B) Western blot analysis of GRP78 cleavage due to SubA2-2-His or SubAB2-2-His treatment of Vero and CaCo-2 cells. Cells were seeded in 12-well plates and incubated with 10 µg/ml SubA2-2-His or a total protein concentration of 10 µg/ml (molar ratio of 1:5) for SubAB2-2-His in FCS-free medium for 6 h at 37 °C. Cells were then solubilized in Lämmli sample buffer and subjected to SDS-PAGE. The substrate status. i.e. GRP78 cleavage, was analyzed by immunoblotting.

**B**

Control

SubA

Vero

0 h

24 h

48 h

Control

SubA

CaCo

0 h

24 h

48 h

SubAB

SubAB


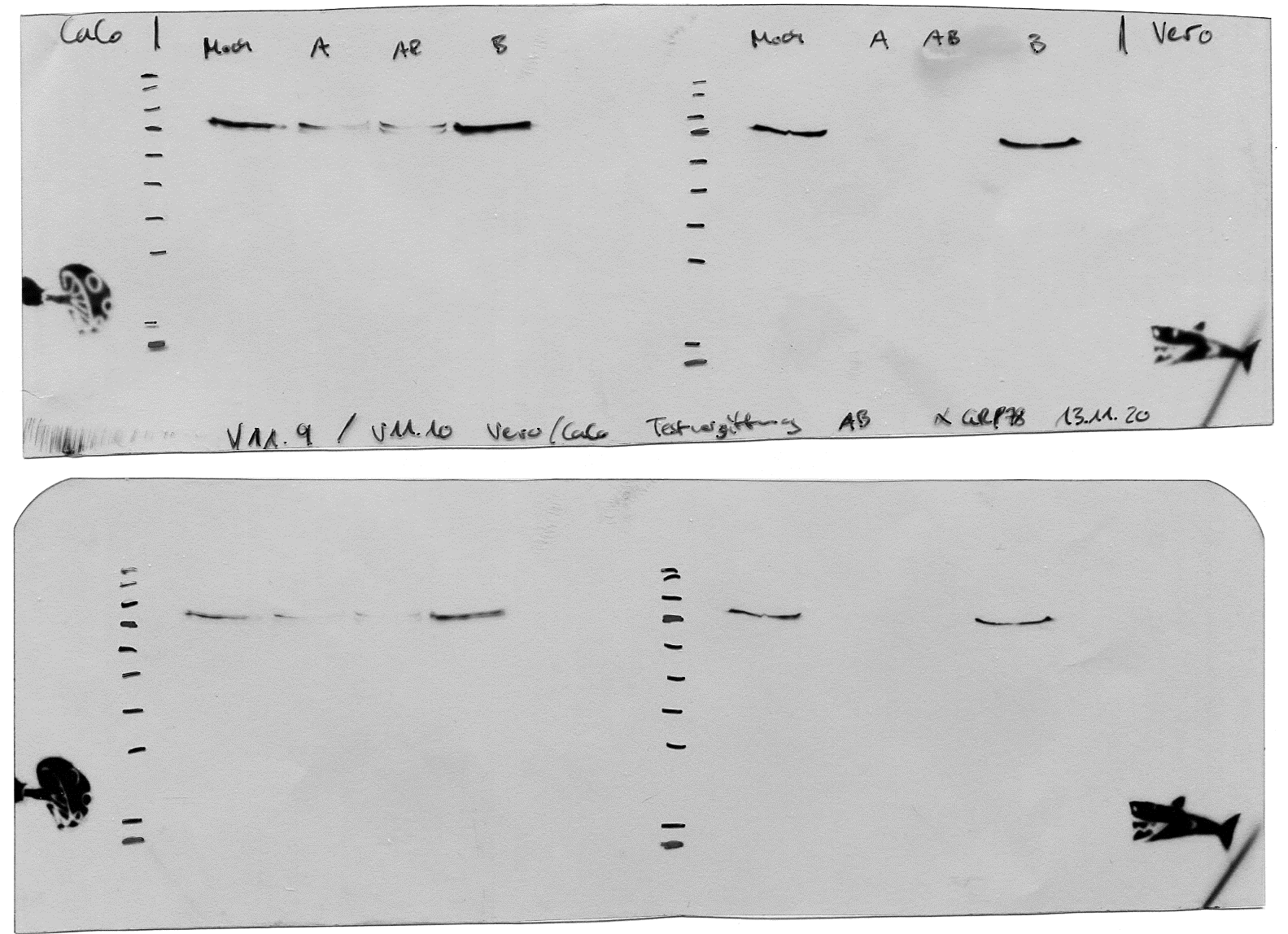


**-**

**+**

**-**

**-**

**+**

**+**

**-**

**+**

SubA

SubB

Vero

95

72

55


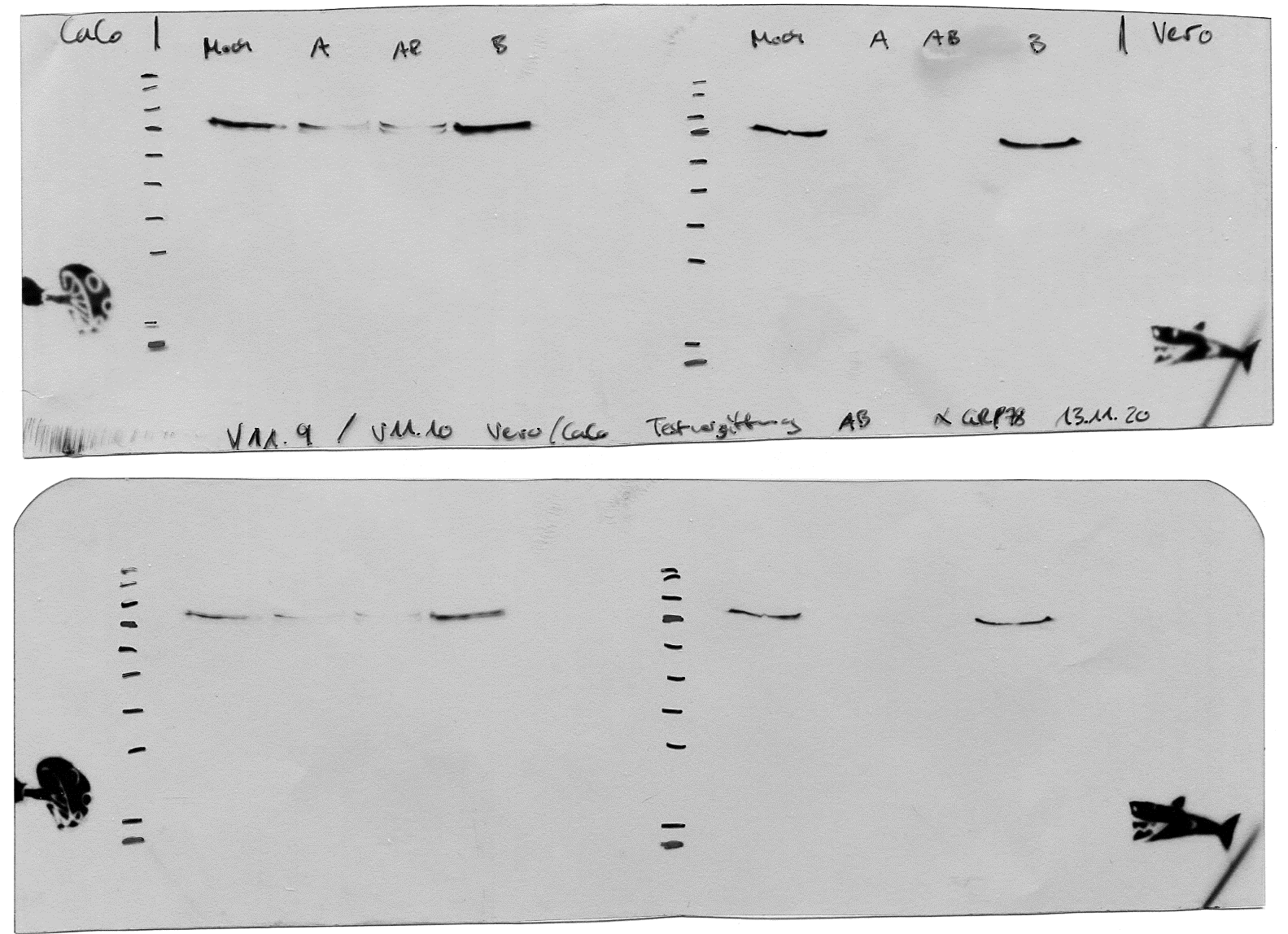


**-**

**+**

**-**

**-**

**+**

**+**

**-**

**+**

SubA

SubB

CaCo

55

72

95

kDA

kDA


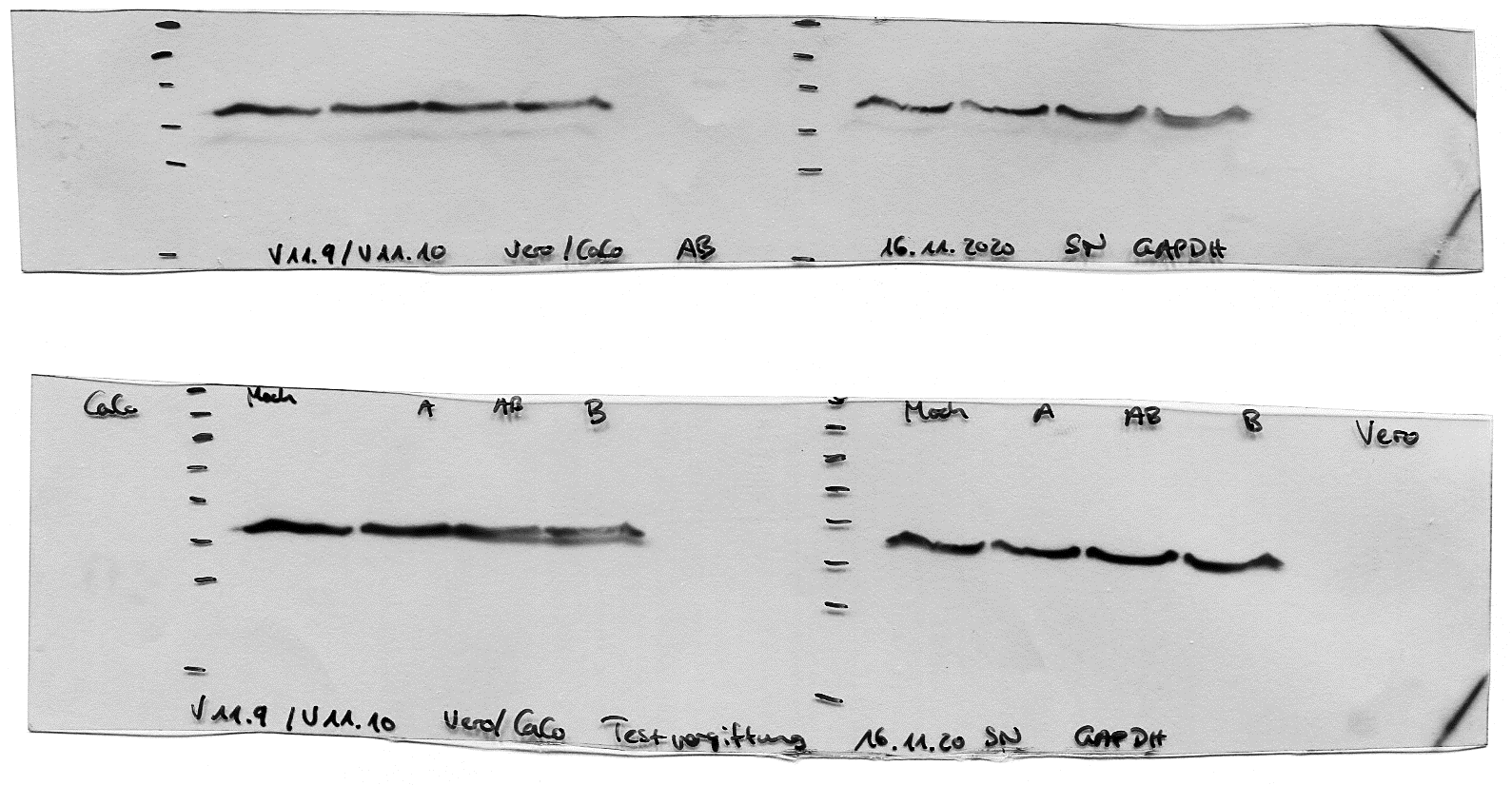


43

34


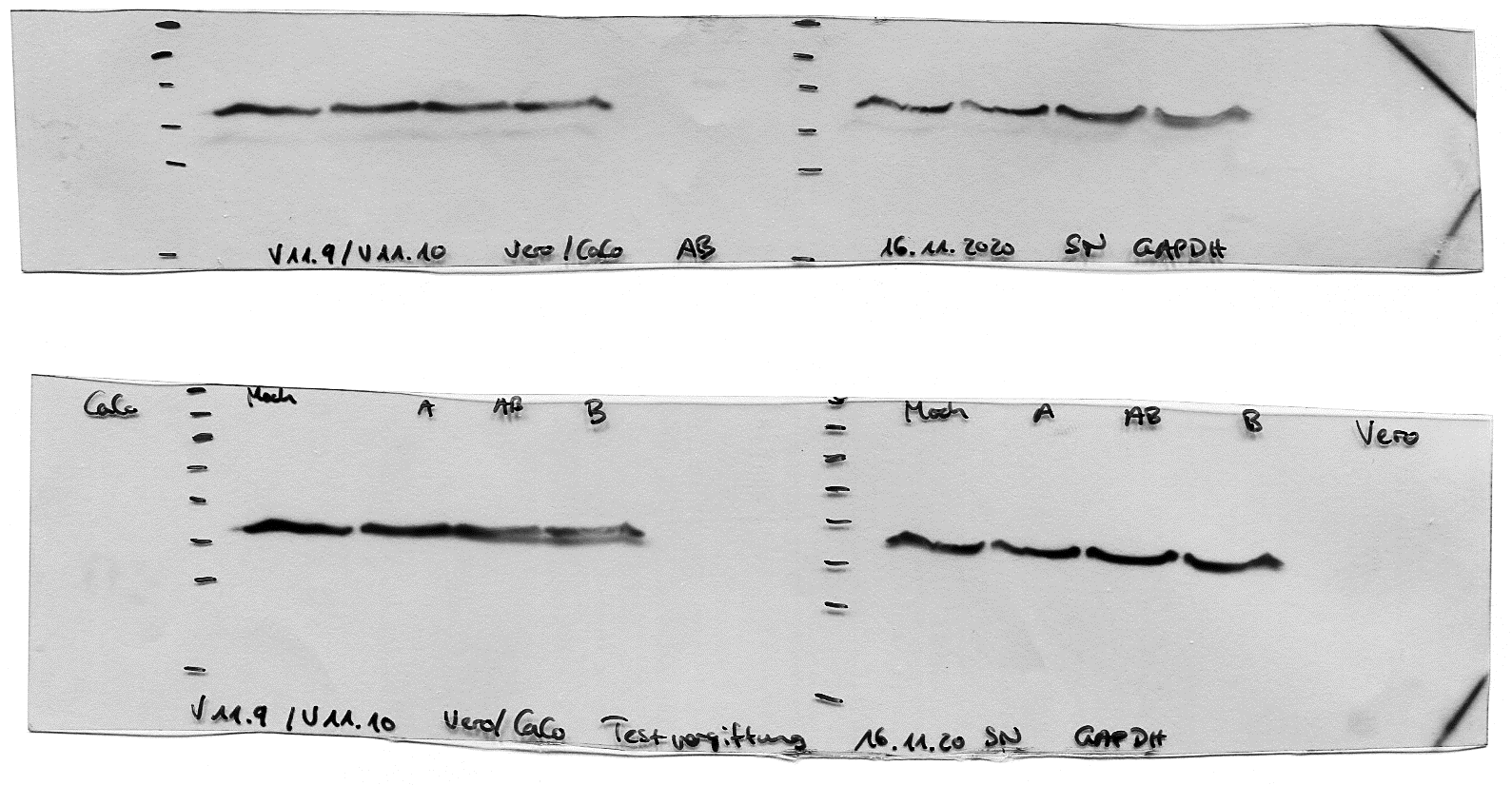


43

34

GRP78

GRP78

GAPDH

GAPDH


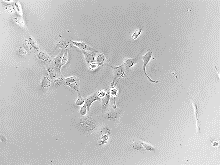

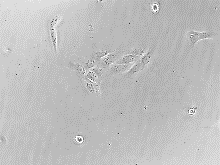

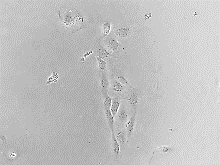

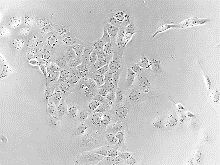

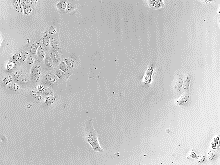

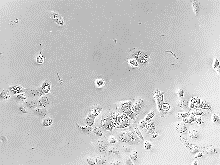

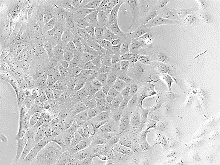

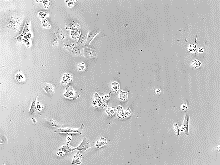

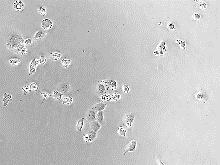

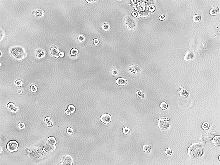

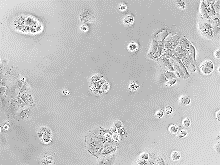

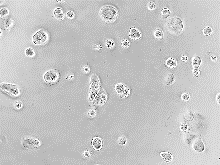

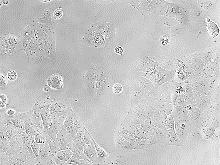

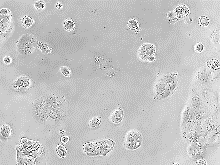

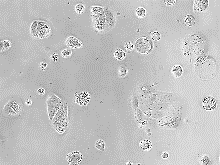

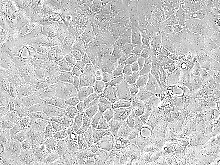

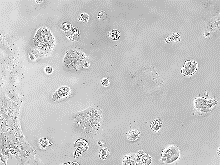

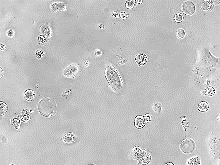


**A**

Figure S 2: HeLa and HCT116 cells were incubated with increasing BFA concentrations as indicated. After 6.5 h of BFA- treatment, cell viability was measured by MTS assay. Values are given as mean ± SD (n=3).


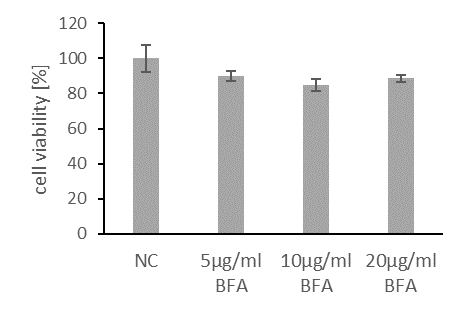


HCT116


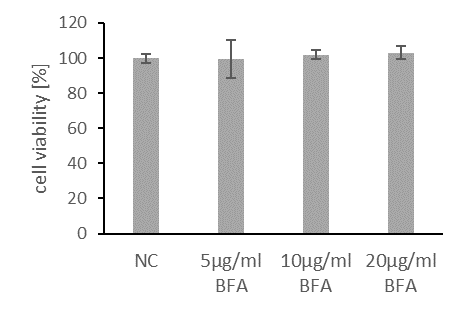


HeLa


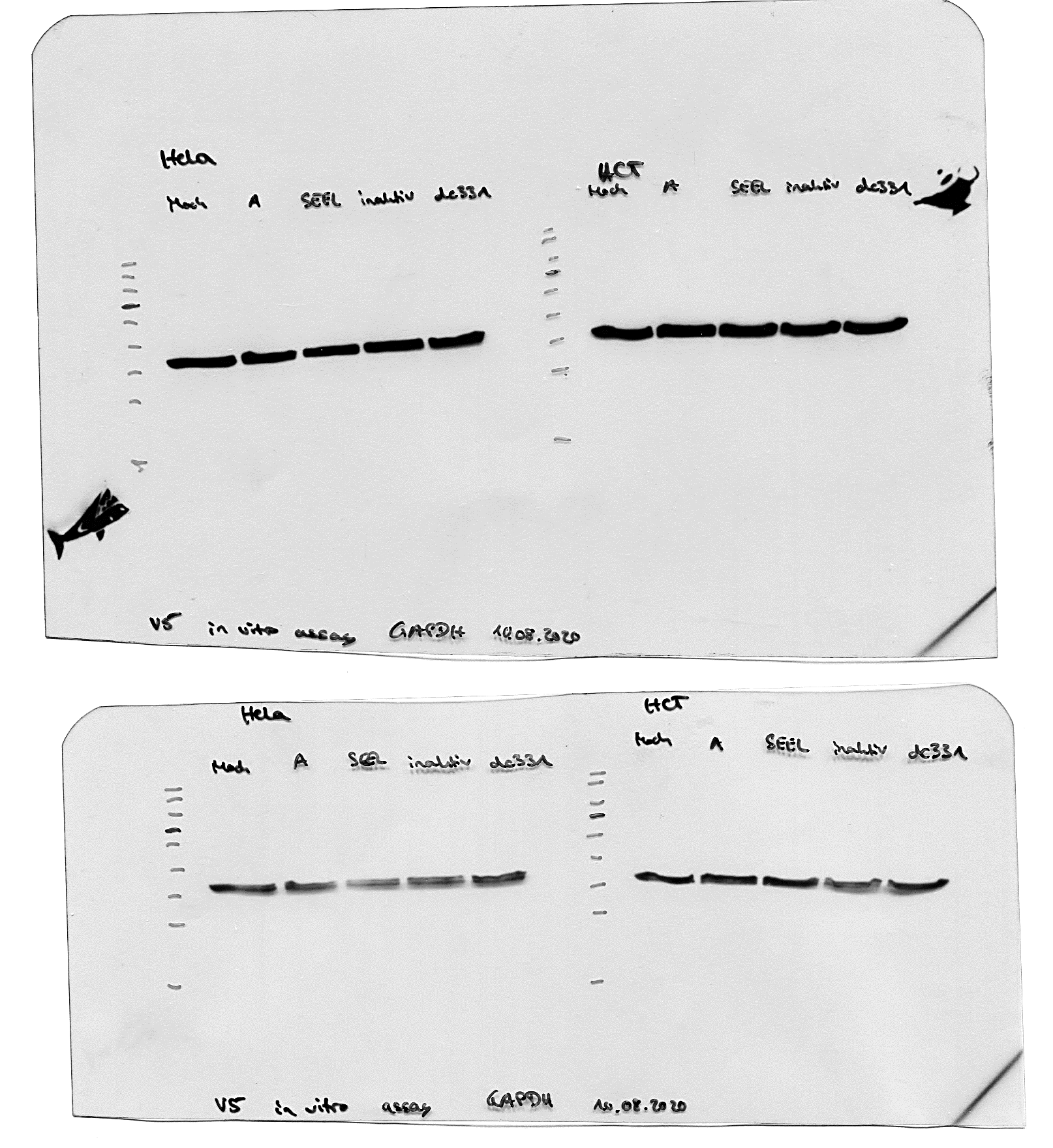


43

34

GAPDH

43

34

GAPDH


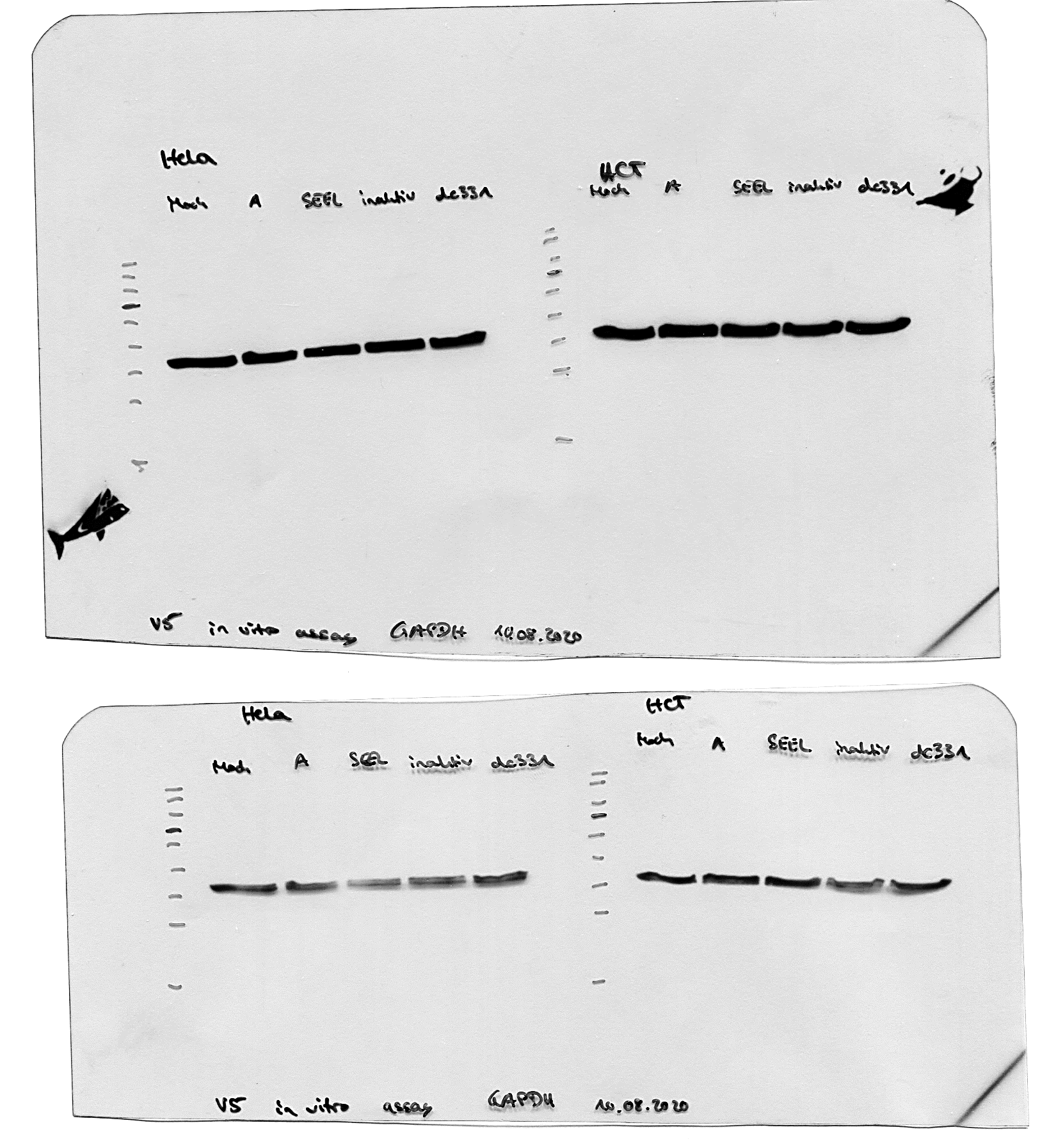


SubA

SubA_S272A_

**-**

**+**

**-**

**+**

**-**

**-**

**-**

**-**

**+**

**-**

**-**

**-**

SubA_ΔC344_

HCT116

95

55

72


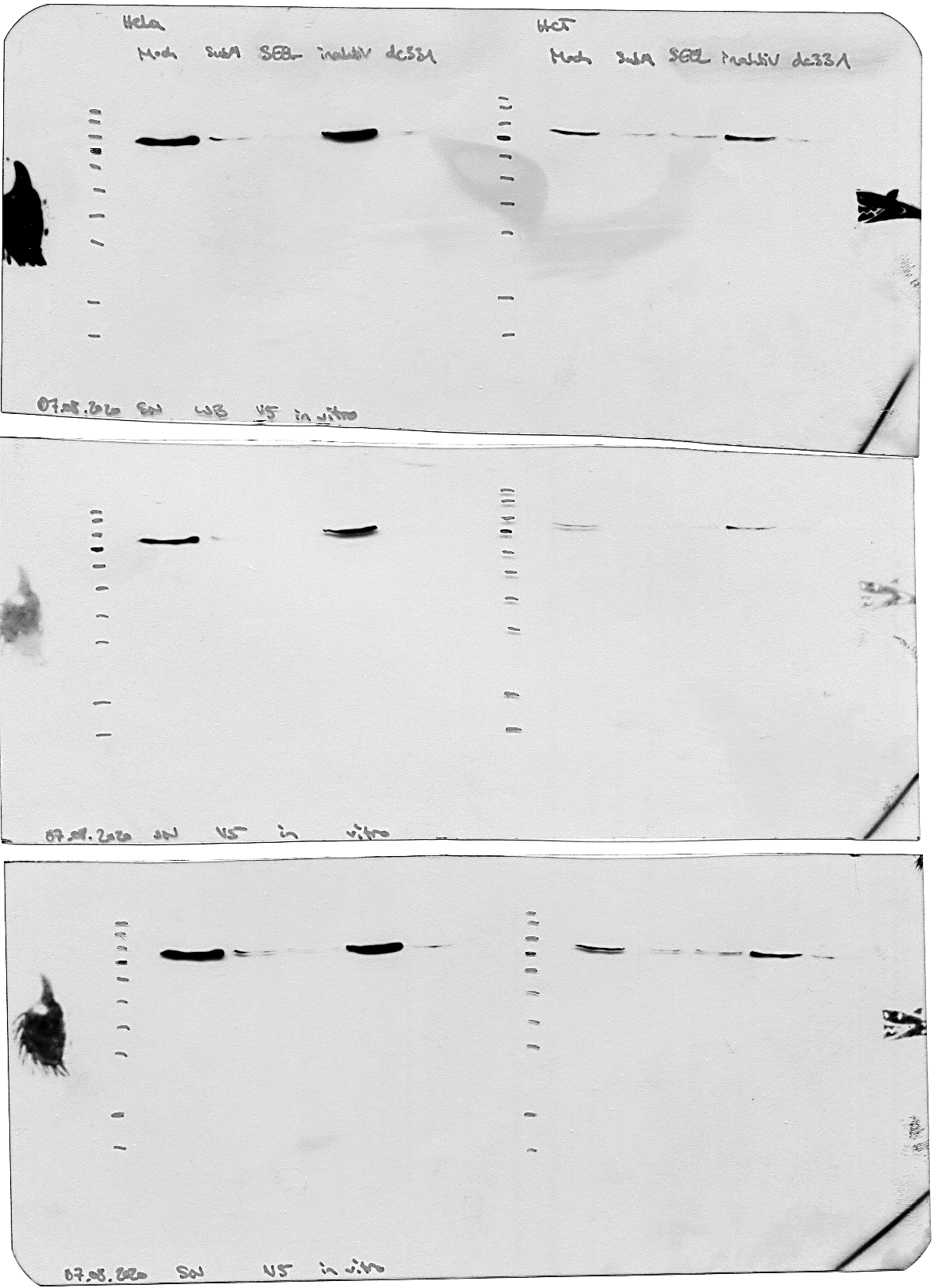


kDa

GRP78

SubA

SubA_S272A_

**-**

**+**

**-**

**+**

**-**

**-**

**-**

**-**

**+**

**-**

**-**

**-**

SubA_ΔC344_

HeLa

95

55

72

kDa


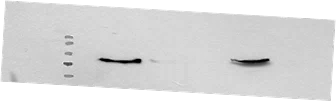


GRP78

**Figure S 3**: Enzyme activity test. Cell lysates were incubated with 10 µg/ml of SubA2-2-His, SubA_ΔC344_2-2-His, or SubA_S272A_2-2-His for 30 min at 37 °C. For control cells were left untreated. GRP78 status was analyzed by Western blotting. Experiment was performed twice.

**Figure S 4:** Flow cytometric analysis of SubA2-2-His and SubA_ΔC344_2-2-His binding to HeLa cells. The relative median fluorescent intensity at 488 nm excitation is shown. Values are given as the mean of three measurements ± standard deviation. HeLa cells were incubated at 4 °C for 30 min with SubA (30 µg/ml of Dylight488-labeled SubA2-2-His) or SubA_∆C344_ (30 µg/ml of Dylight488-labeled SubA_∆C344_2-2-His) or left untreated for control (NC).
